# Supplementary material for: The Crossroads of Precision Medicine and Therapeutic Decision-Making: Use of an Analytical Computational Platform to Predict Response to Cancer Treatments
Source: Cancers (Basel). 2020 Jan 9;12(1):166. doi: 10.3390/cancers12010166 (PMC7017109; doi:10.3390/cancers12010166)
Supplement: Supplementary file 1 [file cancers-12-00166-s001.pdf]

Supporting Information Table 1 – List of publications included in the analysis.

Year of publication	Reference	Number of patients	Case number(s)	Number of regimens
2010	Godbert et al.[i]	1	12	3
	Wagner et al.[ii]	3	29 30,32	5
2011	Villarroel et al.[iii]	1	24	3
2012	Garrido-Laguna et al.[iv]	1	33	1
2013	Falchook et al.[v]	1	14	3
	Artioli et al.[vi]	1	54	5
2014	Falchook et al.[vii]	1	2	4
	Hagemann et al.[viii]	1	7	4
	Loaiza-Bonilla et al.[ix]	1	18	2
	Voss et al.[x]	4	42,43,44,53	8
	Wagle et al.[xi]	1	61	2
	Subbiah et al.[xii]	1	63	4
2015	Wheler et al.[xiii]	10	10,25,26,27,50,55,56,60,65,66	30
	Davar et al.[xiv]	1	13	3
	Mitsis et al.[xv]	1	15	2
	Steenbruggen et al.[xvi]	1	16	2
	Subbiah et al.[xvii]	1	23	2
	VanderWeele et al.[xviii]	1	36	1
	Palma et al.[xix]	1	37	4
	Wheler et al.[xx]	3	49,51,57	17
	Knoechel et al.[xxi]	1	62	1
	Van Allen et al.[xxii]	1	64	1
	Kadakia et al.[xxiii]	1	68	2
2016	Cheng et al.[xxiv]	3	3,8,40	13
	Ikeda et al.[xxv]	1	5	5
	Sharabi et al.[xxvi]	1	17	2
	Mehnert et al.[xxvii]	1	21	1
	Colton et al.[xxviii]	1	22	1
	Ravi et al.[xxix]	1	34	4
	Kieler et al.[xxx]	1	45	3
	Lim et al.[xxxi]	1	52	2
	Loaiza-Bonilla et al.[xxxii]	1	59	2
	Biswas et al.[xxxiii]	1	67	14
	Pietanza et al.[xxxiv]	1	69	1
	Wen et al.[xxxv]	1	70	1
2017	Lheureux et al.[xxxvi]	3	1,41,47	7
	Gibson et al.[xxxvii]	1	4	4
	Kollipara et al.[xxxviii]	1	6	4
	Knepper et al.[xxxix]	1	9	3
	Dos Santos Fernandes et al.[xl]	1	11	3
	Wilkes et al.[xli]	1	19	1
	George et al.[xlii]	1	20	1
	Wander et al.[xliii]	1	28	3
	Patel et al.[xliv]	1	31	1
	Seim et al.[xlv]	1	35	2
	Parachoniak et al.[xlvi]	1	38	6
	Rodríguez-Moreno et al.[xlvii]	1	39	3
	Sorscher et al.[xlviii]	1	46	2
	Pishvaian et al.[xlix]	1	48	1
	Korphaisarn et al.[l]	1	58	3

Supporting Information Table 2 – Description of the 70 exceptional responders reviewed.

Case number	Age	Sex	Diagnosis	Stage/TNM class	Altered biomarkers	Treatment lines & responses (best response, tumor change, time to treatment failure in months)
**1**	49	F	High-grade serous ovarian cancer	M1	BRCA1, TMB high	[cisplatin + paclitaxel (SD, TTF=6mo)] [carboplatin + gemcitabine (SD, TTF=8mo)] [cisplatin + paclitaxel (SD, TTF=8mo)] [olaparib (PR, TTF=41mo)]
**2**	55	M	High-grade salivary duct carcinoma	M1	ERBB2	[lapatinib + trastuzumab + bevacizumab (PR, TTF=25+mo)] [cisplatin + docetaxel (PD)] [carboplatin + docetaxel + trastuzumab (PR)] [zoledronic acid + trastuzumab +/- paclitaxel (SD, TTF=14mo)]
**3**	70	M	Prostate adenocarcinoma	M1	BRCA2	[androgen deprivation (SD, TTF=6mo)] [docetaxel + carboplatin (PR, TTF=24+mo)] [cisplatin + etoposide (SD, TTF=6mo)] [paclitaxel (SD, TTF=6mo)]
**4**	62	F	High-grade anaplastic thyroid carcinoma		BRAF, PIK3CA	[dabrafenib + trametinib + everolimus (PR, TTF=2mo)] [carboplatin + paclitaxel (PD, TTF=1mo)] [everolimus (PD)] [dabrafenib + trametinib (PD, TTF=1.5mo)]
**5**	58	M	Basal cell carcinoma	M1	CDKN1A, CDKN2A, CTNNA1, FLT1, JAK2, LRP1B, MLL2, NOTCH1, PDGFRA, PD-L1, PD-L2, PIK3R2, PTCH1, SLIT2, SMARCA4, TERT, TMB high	[nivolumab (PR, TTF=18+mo)] [vismodegib (PD, TTF=2mo)] [cisplatin + paclitaxel (PR, TTF=2mo)] [sonidegib + buparlisib (PD, TTF=1.5mo)] [paclitaxel + vismodegib (PD, TTF=2mo)]
**6**	62	M	Poorly-differentiated thyroid papillary adenocarcinoma	T2N0M0	BIRC5, BRAF, PD-L1, TUBB3, TYMP	[doxorubicin + cisplatin (PD)] [paclitaxel (PD)] [vemurafenib (PD)] [nivolumab (CR, TTF=20+mo)]
**7**	55	F	Poorly differentiated squamous cell carcinoma of thymus	M1	KIT	[imatinib (SD, -20%, TTF=12+mo)] [paclitaxel + carboplatin (PR, -50%, TTF=4mo)] [pemetrexed (PD)] [gemcitabine (PD, TTF=3mo)]

**8**	66	M	Prostate adenocarcinoma	M1	BRCA2	[docetaxel (SD, TTF=4mo)] [abiraterone (PD, TTF=1mo)] [enzalutamide (PD, TTF=1mo)] [docetaxel + carboplatin (SD, TTF=12mo)]
**9**	60	M	Basal squamous cell carcinoma		KDR	[pazopanib (CR, TTF=6mo)] [vismodegib (CR, TTF=5mo)] [carboplatin + paclitaxel (PD)]
**10**			Melanoma		BRAF, NF1	[dabrafenib + trametinib (CR, -100%, TTF=6mo)] [unknown/unmatched (PD)] [unknown/unmatched (PD)]
**11**	50	F	Gastric cancer		MTB high	[epirubicin + oxaliplatin + capecitabine (PD)] [fluorouracil + oxaliplatin (SD)] [pembrolizumab (CR)]
**12**	71	F	Anaplastic thyroid cancer	IVB / pT4aN1bM1	ALK	[crizotinib (PR, -90%, TTF=24+mo)] [cisplatin + etoposide (SD, TTF=6mo)] [carboplatin + etoposide (PD, TTF=3mo)]
**13**	77	F	Lung adenocarcinoma	M1	KRAS, PD-L1, TP53	[carboplatin + pemetrexed (PD)] [docetaxel + FAK inhibitor + gemcitabine + pemetrexed (PD)] [nivolumab (PR)]
**14**	53	F	Lung adenocarcinoma	M1	ERBB2	[lapatinib + trastuzumab + bevacizumab (PR, TTF=7+mo)] [cisplatin+docetaxel (SD)] [carboplatin+pemetrexed (SD)]
**15**	69	M	High-grade sarcoma abdominal wall		BRAF	[gemcitabine + docetaxel (PD)] [dabrafenib + trametinib (CR, TTF=14mo)]
**16**	32	F	Breast ductal carcinoma	M1	BRCA1	[fluorouracil + epirubicin + cyclophosphamide (PD)] [thiothepa + cyclophosphamide + carboplatin (CR)]
**17**	48	F	High-grade large-cell neuroendocrine cervical carcinoma	M1	"ABL2, ATRX, BLM, CTNNB1, FBXW7, FGF6, JAK1, MEN1, MLL2, MLL3, MSH2, MSI high, NOTCH1, NRAS, PD-L1, PIK3CA, PREX2, PTEN, QKI, SETD2, SMARCA4, STK11, TET2, TMB high, octreotide scan positive	[nivolumab + octreotide (PR, -95%, TTF=17+mo)] [cisplatin + etoposide (PD)]
**18**	47	F	Poorly differentiated intrahepatic cholangiocarcinoma	"		[dabrafenib + trametinib (PR, TTF=8.5+mo)] [fluorouracil (PD)]
**19**		M	Small-cell neuroendocrine prostate cancer	M1	BRAF	[cisplatin (CR)]

**20**		F	Uterine leiomyosarcoma		FANCA	[pembrolizumab (PR, TTF=9mo)]
**21**	53	F	High-grade endometrioid adenocarcinoma	M1	MYC, PD-L1, PD-L2, PTEN, TP53	[pembrolizumab (PR, TTF=14mo)]
**22**	53	M	Stomach adenocarcinoma	IB / pT1N0	PD-L1, POLE, TMB high	[fluorouracil + oxaliplatin (PR, TTF=4+mo)]
**23**	74	F	lung adenocarcinoma		PALB2	[carboplatin + pemetrexed + zoledronic acid + docetaxel (PD)] [vandetanib + everolimus (PR)]
**24**	61	M	Ductal pancreatic adenocarcinoma	M1	AKT2, RET	[mitomycin C (PR, TTF=22mo)] [cisplatin (PR, TTF=36+mo)] [gemcitabine (SD, TTF=4mo)]
**25**			Melanoma	pT3N1M0	PALB2	[dabrafenib (CR, -100%, TTF=29+mo)] [unknown/unmatched (PD)] [unknown/unmatched (PD)]
**26**			Melanoma		BRAF	[dabrafenib (CR, -100%, TTF=24+mo)] [unknown/unmatched (PD)] [unknown/unmatched (PD)]
**27**			Melanoma		BRAF	[trametinib (PR, -53%, TTF=7mo)] [unknown/unmatched (PD)] [unknown/unmatched (PD)]
**28**	44	F	Therapy-related myeloid neoplasm, progressing in acute myeloid leukemia		BRAF	[dabrafenib + trametinib (PR, TTF=1mo)] [decitabine (PD)] [cytarabine + topotecan (PD)]
**29**	65	M	Perivascular epithelioid cell tumor		BRAF, KRAS, MLLT3-KMT2A	[sirolimus (PR, TTF=16+mo)] [MET inhibitor (PD)]
**30**	70	M	Perivascular epithelioid cell tumor		mTORC1, TSC2	[sirolimus (PR, -40%, TTF=10mo)] [sunitinib (PD, TTF=1.5mo)]
**31**	50	F	Cutaneous Castleman's disease		mTORC1, TSC2	[siltuximab (CR, TTF=84mo)]
**32**	61	F	Perivascular epithelioid cell tumor		JAK1	[sirolimus (PR, TTF=7mo)]
**33**			Pancreatic cancer		mTORC1, TSC1, TSC2	[trametinib (PR, TTF=10mo)]

**34**			Angiosarcoma		KRAS	[paclitaxel (SD, TTF=12mo)] [sorafenib (PD, TTF=3mo)] [pazopanib (SD, TTF=6mo)] [gemcitabine + docetaxel (PD, TTF=2mo)]
**35**	60	M	Squamous cell carcinoma		ARID1A, MCL1, TP53, VEGFR2, VEGFR3	[lapatinib (SD, TTF=3mo)] [lapatinib + fluorouracil + irinotecan (CR, TTF=60+mo)]
**36**	82	M	Prostate adenocarcinoma	T2N2M1	"HPV positive	[carboplatin + gemcitabine + veliparib (CR)]
**37**	67	F	high-grade papillary urothelial carcinoma	ERBB2, ERCC1, TOPO1, TOP2A, TYMS"		[methotrexate + vinblastine + doxorubicin + cisplatin (PD)] [doxorubicin + iphosphamide (PD)] [paclitaxel + methotrexate + gemcitabine + cyclophosmpahmie (PD)] [pazopanib (PR)]
**38**	49	F	Breast ductal carcinoma / undifferenciated carcinoma	M1	AR, BRCA2, TMPRSS2-ERG, TP53	[exemestane + everolimus (CR, TTF=14+mo)] [carboplatin + gemcitabine (PR, TTF=9mo)] [anastrozole + fulvestrant (PD, TTF=3mo)] [anastrozole + fulvestrant + capecitabine (CR, TTF=12mo)] [paclitaxel (SD, TTF=8mo)] [megestrol + gencitabine + carboplatin (SD, TTF=7mo)]
**39**	57	F	Clear cell renal carcinoma	M1	CCND1, FGF19, FGFR3	[sunitinib (PD)] [sorafenib (PD)] [temsirolimus (CR)]
**40**	53	M	Prostate adenocarcinoma	III / T1bN1M1	ER, PR, STK11	[androgen deprivation (SD, TTF=36mo)] [abiraterone (SD, TTF=18mo)] [enzalutamide (SD, TTF=12mo)] [docetaxel (PD, TTF=2mo)] [carboplatin + doxorubicin (PR, TTF=6mo)]
**41**	51	F	High-grade serous ovarian cancer	IV	BAP1, MTOR, VHL1	[olaparib (CR, TTF=77mo)] [carboplatin + paclitaxel (SD, TTF=4mo)]
**42**	73	F	Renal clear cell cancer	M1	BRCA2	[temsirolimus (SD, TTF=34mo)] [sunitinib (SD, TTF=3mo)]
**43**	58	F	Renal clear cell cancer		BRCA2, TP53	[temsirolimus (SD, TTF=27mo)] [sunitinib (SD, TTF=14mo)]
**44**	66	M	Renal clear cell cancer	M1	TP53, TSC1, VHL	[everolimus (SD, TTF=20mo)] [sunitinib (SD, TTF=5mo)]
**45**	42	F	Colorectal cancer	M1	PBRM1, TSC1, VHL	[pembrolizumab (PR, -30%, TTF=9+mo)] [capecitabine + oxaliplatin (SD, TTF=9mo)] [fluorouracil + irinotecan (SD, TTF=9mo)]
**46**	83	F	Colon adenocarcinoma		BAP1, MTOR, TSC1, VHL	[nivolumab (PR, TTF=6+mo)] [fluorouracil + oxaliplatin (SD, TTF=7mo)]

**47**	65	F	High-grade serous ovarian cancer	III / M1	KRAS, MLH1, PMS2	[olaparib (CR, TTF=86mo)]
**48**	63	F	Pancreatic cancer	IIIB / T4bN1M1	APC, CDK8, EPHA3, ETV6, FLT3, KRAS, PD-L1, PD-L2, STAG2, TMB intermediate, TP53	[fluorouracil + oxaliplatin + veliparib (PR, TTF=15mo)]
**49**	48	F	Breast ductal carcinoma		BRCA1, TP53	[anastrozole + everolimus (PR, -38%, TTF=14mo)] [anastrozole + everolimus + fulvestrant (PR, TTF=5+mo)] [tamoxifen (PD, TTF=2mo)] [paclitaxel + bevacizumab (PD)] [vinolrebine (PD)] [fulvestrant (PD, TTF=3mo)] [ixabepilone (PD)] [docetaxel (SD, TTF=9mo)] [docetaxel + doxorubicin + cyclophosphamide (PD, TTF=3mo)]
**50**			Melanoma	M1	BRCA2, KRAS, TP53	[dabrafenib + trametinib (PR, -34%, TTF=11mo)] [unknown/unmatched (PD)] [unknown/unmatched (PD)]
**51**	38	F	Breast ductal carcinoma	M1	CCND1, ER, FGFR1, MYC, PIK3CA, PIK3R1, PR	[anastrozole + everolimus (PR, -56%, TTF=11mo)] [paclitaxel (PD, TTF=3mo)] [fluorouracil + doxorubicin + cyclophosphamide (PD, TTF=1mo)] [tamoxifen (PD, TTF=3mo)] [capecitabine (PD, TTF=3mo)]
**52**	51	M	Ductal adenocarcinoma of the lacrimal gland		APC, BRAF, NRAS, PTEN, RB1	[? (PD)] [everolimus (PR)]
**53**	60	F	Renal clear cell cancer	M1	CCND1, ER, FGFR1, PR, PRKDC, PTEN	[temsirolimus (SD, TTF=28mo)] [sunitinib (SD, TTF=11mo)]
**54**	29	F	Breast ductal carcinoma	M1	NF1, TP53	[cisplatin + nab-paclitaxel + zoledronic acid (CR, TTF=4.5mo)] [tamoxifen + zoledronic acid (SD, TTF=7mo)] [fulvestrant (SD, TTF=7mo)] [fluorouracil + epirubicin + cyclophosphamide then docetaxel (SD, TTF=54 for 2 linesmo)] [fluorouracil + epirubicin + cyclophosphamide then triptorelin + tamoxifen (SD, TTF=54mo for 2 lines)]
**55**			Melanoma	M1	PBRM1, VHL	[dabrafenib (CR, -100%, TTF=27+mo)] [unknown/unmatched (PD)] [unknown/unmatched (PD)]
**56**			Melanoma	pT2N2aM1	BRCA2, ER, PR, TP53	[dabrafenib (PR, -48%, TTF=6mo)] [unknown/unmatched (PD)] [unknown/unmatched (PD)]

**57**	44	F	Breast ductal carcinoma		AURKA, BRAF	[anastrozole + everolimus (CR, -100%, TTF=9+mo)] [tamoxifen + zoledronic acid (SD, TTF=6mo)] [letrozole (SD, TTF=4mo)]
**58**	53	M	Rectal cancer		BRAF, MITF	[fluorouracil + oxaliplatin (SD, TTF=4mo)] [fluorouracil + oxaliplatin + irinotecan (SD, TTF=8mo)] [regorafenib (PR, TTF=20mo)]
**59**	84	F	High-grade colorectal adenocarcinoma	M1	CCNE1, ER, IRS2, MCL1, TP53	[fluorouracil + bevacizumab (PD, TTF=3mo)] [regorafenib (low dose) (PR, TTF=9+mo)]
**60**			Melanoma	M1	APC, FLT4, KRAS, PPP1R3A, TP53	[dabrafenib (PR, -54%, TTF=3mo)] [unknown/unmatched (PD)] [unknown/unmatched (PD)]
**61**	57	F	Anaplastic thyroid cancer	pT4bN1bM1	APC, ERBB2, KDR, KRAS	[everolimus (PR, TTF=18mo)] [carboplatin + paclitaxel (PD, TTF=3mo)]
**62**	53	M	Early T-cell progenitor acute lymphoblastic leukemia		BRAF, CDKN2A, MYC	[BMS-906024 (pan-Notch inhibitor) + dexamethasone (CR, TTF=19mo)]
**63**	52	M	Testicular germ cell tumor	N1M1	FLCN, PTEN, TMB high, TSC2	[sunitinib (PR, TTF=17mo)] [bleomycin + ifosfamide + etoposide + cisplatin (SD, TTF=4mo)] [paclitaxel + ifosfamide + cisplatin (PD, TTF=2mo)] [actinomycin D + cyclophosphamide + etoposide (PD, TTF=3mo)]
**64**	32	M	Head and neck squamous cell carcinoma		CSF3R, DNMT3A, NOTCH1, PTPN11	[erlotinib (CR)]
**65**			Melanoma	N1M1	AKT1, EGFR, KRAS, PTEN, RET	[vemurafenib (PR, -38%, TTF=8mo)] [unknown/unmatched (PD)] [unknown/unmatched (PD)]
**66**			Melanoma	IVA / T1N2cM0	ABL2, ARID1A, MAPK1, TP53	[dabrafenib (PR, -75%, TTF=4mo)] [unknown/unmatched (PD)] [unknown/unmatched (PD)]
**67**	50	M	Lung adenocarcinoma		BRAF, CDKN2A, CDKN2B, NF1, PAX5	[carboplatin + paclitaxel + bevacizumab (SD, TTF=6mo)] [erlotinib (PD, TTF=3mo)] [dacomitinib (SD, TTF=7mo)] [trastuzumab (PD, TTF=3mo)] [trastuzumab + vinolrebine (SD, TTF=18mo)] [lapatinib (SD, TTF=4mo)] [crizotinib (PD, TTF=1mo)] [lapatinib + capecitabine (SD, TTF=10mo)] [lapatinib + nab-paclitaxel (PD, TTF=3mo)] [lapatinib + pemetrexed (PD, TTF=3mo)] [pertuzumab + trastuzumab +

						docetaxel (SD, TTF=12mo)] [trastuzumab emtansine (PD, TTF=3mo)] [pertuzumab + trastuzumab + afatinib (SD, TTF=4mo)] [trastuzumab + vinolrebine + everolimus (SD, TTF=12mo)]
**68**	47	M	Prostate adenocarcinoma		ATM, BRAF, CDKN2A, CDKN2B, MET	[anti-androgen + leuprolide (PR)] [etoposide + carboplatin + leuprolide (PD)]
**69**			Small cell lung cancer	IV	ABL1, CDK12, CIITA, CTSG, DNMT3A, ELN, ERBB2, FAM20C, HIP1, IL2, MET, MLL3, NF2, PDE4DIP, PHKG1, RB1, ROS1, SBDS, TP53	[sonidegib + etoposide + cisplatin (PR, TTF=27+mo)]
**70**		M	Head and neck squamous cell carcinoma	M1	AR, MAP2K4, MYCL, PTEN, RB1, SMAD4, TP53,	[erlotinib (PR, TTF=24+mo)]

Abbreviations: CR = complete response; F = female; M = male; mo = month(s); MMR = mismatch repair; MSI = microsatellite instability; PD = progressive disease; PR = partial response; SD = stable disease; TMB = tumor mutation burden; TTF = time to treatment failure.

[i] Godbert Y, Henriques de Figueiredo B, Bonichon F, Chibon F, Hostein I, Pérot G, Dupin C, Daubech A, Belleannée G, Gros A, Italiano A, Soubeyran I. Remarkable Response to Crizotinib in Woman With Anaplastic Lymphoma Kinase-Rearranged Anaplastic Thyroid Carcinoma. J Clin Oncol. 2015 Jul 10;33(20):e84-7. doi: 10.1200/JCO.2013.49.6596.

ii Wagner AJ, Malinowska-Kolodziej I, Morgan JA, et al. Clinical Activity of mTOR Inhibition With Sirolimus in Malignant Perivascular Epithelioid Cell Tumors: Targeting the Pathogenic Activation of mTORC1 in Tumors. Journal of Clinical Oncology. 2010;28(5):835-840. doi:10.1200/JCO.2009.25.2981.

iii Villarroel MC, Rajeshkumar NV, Garrido-Laguna I, et al. Personalizing cancer treatment in the age of global genomic analyses: PALB2 gene mutations and the response to DNA damaging agents in pancreatic cancer. Molecular Cancer Therapeutics. 2011;10(1):3-8. doi:10.1158/1535-7163.MCT-10-0893.

iv Garrido-Laguna I, Tometich D, Hu N, et al. N of 1 case reports of exceptional responders accrued from pancreatic cancer patients enrolled in first-in-man studies from 2002 through 2012. Oncoscience. 2015;2(3):285-293.

v Falchook GS, Janku F, Tsao AS, Bastida CC, Stewart DJ, Kurzrock R. Non-Small Cell Lung Cancer with HER2 Exon 20 Mutation: Regression with Dual HER2 Inhibition and Anti-VEGF Combination Treatment. Journal of thoracic oncology : official publication of the International Association for the Study of Lung Cancer. 2013;8(2):e19-e20. doi:10.1097/JTO.0b013e31827ce38e.

vi Artioli G, Azzarello G, Meggiolaro F, Wabersich J, Borgato L, Bari M. Case Report: Complete Clinical Response of a Young Patient with a BRCA 2 Positive Metastatic Breast Cancer. Advances in Breast Cancer Research, Vol.2 No.3(2013). DOI:10.4236/abcr.2013.23010

vii Falchook GS, Lippman SM, Bastida CC, Kurzrock R. HER2-Amplified Salivary Duct Carcinoma: Regression with Dual HER2 Inhibition and Anti-VEGF Combination Treatment. Head & neck. 2014;36(3):E25-E27. doi:10.1002/hed.23429.

viii Hagemann IS, Govindan R, Javidan-Nejad C, Pfeifer JD, Cottrell CE. Stabilization of disease after targeted therapy in a thymic carcinoma with KIT mutation detected by clinical next-generation sequencing. J Thorac Oncol. 2014 Feb;9(2):e12-6. doi: 10.1097/JTO.0b013e3182a7d22e.

ix Loaiza-Bonilla A, Clayton E, Furth E, O'Hara M, Morrissette J. Dramatic response to dabrafenib and trametinib combination in a BRAF V600E-mutated cholangiocarcinoma: implementation of a molecular tumour board and next-generation sequencing for personalized medicine. ecancermedicalscience. 2014;8:479. doi:10.3332/ecancer.2014.479

x Voss MH, Hakimi AA, Pham CG, et al. Tumor Genetic Analyses of Patients with Metastatic Renal Cell Carcinoma and Extended Benefit from mTOR Inhibitor Therapy. Clinical cancer research : an official journal of the American Association for Cancer Research. 2014;20(7):1955-1964. doi:10.1158/1078-0432.CCR-13-2345.

xi Wagle N, Grabiner BC, Van Allen EM, et al. Response and Acquired Resistance to Everolimus in Anaplastic Thyroid Cancer. The New England journal of medicine. 2014;371(15):1426-1433. doi:10.1056/NEJMoa1403352.

xii Subbiah V, Meric-Bernstam F, Mills GB, et al. Next generation sequencing analysis of platinum refractory advanced germ cell tumor sensitive to Sunitinib (Sutent®) a VEGFR2/PDGFRβ/c-kit/ FLT3/RET/CSF1R inhibitor in a phase II trial. Journal of Hematology & Oncology. 2014;7:52. doi:10.1186/s13045-014-0052-x.

xiii Wheler J, Yelensky R, Falchook G, et al. Next generation sequencing of exceptional responders with BRAF-mutant melanoma: implications for sensitivity and resistance. BMC Cancer. 2015;15:61. doi:10.1186/s12885-015-1029-z.

xiv Davar D, Socinski MA, Dacic S, Burns TF. Near complete response after single dose of nivolumab in patient with advanced heavily pre-treated KRAS mutant pulmonary adenocarcinoma. Exp Hematol Oncol. 2015 Dec 14;4:34. doi: 10.1186/s40164-015-0029-7.

xv Mitsis D, Opyrchal M, Zhao Y, Kane Iii JM, Cheney R, Salerno KE. Exceptional Clinical Response to BRAF-Targeted Therapy in a Patient with Metastatic Sarcoma. Cureus. 2015 Dec 28;7(12):e439. doi: 10.7759/cureus.439.

xvi Steenbruggen TG, Linn SC(2), Rodenhuis S, Sonke GS. Ongoing Remission Nineteen Years after High-dose Chemotherapy for Oligometastatic Breast Cancer; What Can We Learn from this Patient? Cureus. 2015 Dec 24;7(12):e433. doi: 10.7759/cureus.433.

xvii Subbiah V, Berry J, Roxas M, Guha-Thakurta N, Subbiah IM, Ali SM, McMahon C, Miller V, Cascone T, Pai S, Tang Z, Heymach JV. Systemic and CNS activity of the RET inhibitor vandetanib combined with the mTOR inhibitor everolimus in KIF5B-RET re-arranged non-small cell lung cancer with brain metastases. Lung Cancer. 2015 Jul;89:76-9. doi: 10.1016/j.lungcan.2015.04.004.

xviii VanderWeele DJ, Paner GP, Fleming GF, Szmulewitz RZ. Sustained Complete Response to Cytotoxic Therapy and the PARP Inhibitor Veliparib in Metastatic Castration-Resistant Prostate Cancer - A Case Report. Front Oncol. 2015 Jul 22;5:169. doi: 10.3389/fonc.2015.00169. eCollection 2015.

xix Palma N, Morris JC, Ali SM, Ross JS, Pal SK. Exceptional Response to Pazopanib in a Patient with Urothelial Carcinoma Harboring FGFR3 Activating Mutation and Amplification. Eur Urol. 2015 Jul;68:168-70. doi: 10.1016/j.eururo.2015.02.023.

xx Wheler JJ, Atkins JT, Janku F, Moulder SL, Yelensky R, Stephens PJ, Kurzrock R. Multiple gene aberrations and breast cancer: lessons from super-responders. BMC Cancer. 2015;15:442. doi:10.1186/s12885-015-1439-y.

xxi Knoechel B, Bhatt A, Pan L, Pedamallu CS, Severson E, Gutierrez A, Dorfman DM, Kuo FC, Kluk M, Kung AL, Zweidler-McKay P, Meyerson M, Blacklow SC, DeAngelo DJ , Aster JC. Complete hematologic response of early T-cell progenitor acute lymphoblastic leukemia to the γ-secretase inhibitor BMS-906024: genetic and epigenetic findings in an outlier case. Cold Spring Harb Mol Case Stud. 2015 Oct;1:a000539. doi: 10.1101/mcs.a000539.

xxii Van Allen EM, Lui VW, Egloff AM, Goetz EM, Li H, Johnson JT, Duvvuri U, Bauman JE, Stransky N, Zeng Y, Gilbert BR, Pendleton KP, Wang L, Chiosea S, Sougnez C, Wagle N, Zhang F, Du Y, Close D, Johnston PA, McKenna A, Carter SL, Golub TR, Getz G, Mills GB, Garraway LA, Grandis JR. Genomic Correlate of Exceptional Erlotinib Response in Head and Neck Squamous Cell Carcinoma. JAMA Oncol. 2015 May;1(2):238-44. doi: 10.1001/jamaoncol.2015.34.

xxiii Kadakia KC, Tomlins SA, Sanghvi SK, Cani AK, Omata K, Hovelson DH, Liu CJ, Cooney KA. Comprehensive serial molecular profiling of an "N of 1" exceptional non-responder with metastatic prostate cancer progressing to small cell carcinoma on treatment. J Hematol Oncol. 2015 Oct 6;8:109. doi: 10.1186/s13045-015-0204-7.

xxiv Cheng HH, Pritchard CC, Boyd T, Nelson PS, Montgomery B. Biallelic Inactivation of BRCA2 in Platinum-sensitive Metastatic Castration-resistant Prostate Cancer. Eur Urol. 2016 Jun;69(6):992-5. doi: 10.1016/j.eururo.2015.11.022.

xxv Ikeda S, Goodman AM, Cohen PR, Jensen TJ, Ellison CK, Frampton G, Miller V, Patel SP, Kurzrock R. Metastatic basal cell carcinoma with amplification of PD-L1: exceptional response to anti-PD1 therapy. NPJ Genom Med. 2016;1. pii: 16037.

xxvi Sharabi A, Kim SS, Kato S, Sanders PD, Patel SP, Sanghvi P, Weihe E, Kurzrock R. Exceptional Response to Nivolumab and Stereotactic Body Radiation Therapy (SBRT) in Neuroendocrine Cervical Carcinoma with High Tumor Mutational Burden: Management Considerations from the Center For Personalized Cancer Therapy at UC San Diego Moores Cancer Center. Oncologist. 2017 Jun;22(6):631-637. doi: 10.1634/theoncologist.2016-0517.

xxvii Mehnert JM, Panda A, Zhong H, Hirshfield K, Damare S, Lane K, Sokol L, Stein MN, Rodriguez-Rodriquez L, Kaufman HL, Ali S, Ross JS, Pavlick DC, Bhanot G, White EP, DiPaola RS, Lovell A, Cheng J, Ganesan S. Immune activation and response to pembrolizumab in POLE-mutant endometrial cancer. J Clin Invest. 2016 Jun 1;126(6):2334-40. doi: 10.1172/JCI84940.

xxviii Colton B, Hartley M, Manning MA, Carroll JE, Xiu J, Smaglo BG, Mikhail S, Salem ME. Exceptional Response to Systemic Therapy in Advanced Metastatic Gastric Cancer: A Case Report. Cureus. 2016 Jan 12;8:e457. doi: 10.7759/cureus.457.

xxix Ravi V, Sanford EM, Wang WL, Ross JS, Ramesh N, Futreal A, Patel S, Stephens PJ, Miller VA, Ali SM. Antitumor Response of VEGFR2- and VEGFR3-Amplified Angiosarcoma to Pazopanib. J Natl Compr Canc Netw. 2016 May;14(5):499-502.

xxx Kieler M, Scheithauer W, Zielinski CC, Chott A, Al-Mukhtar A, Prager GW. Case report: impressive response to pembrolizumab in a patient with mismatch-repair deficient metastasized colorectal cancer and bulky disease. ESMO Open. 2016;1(6):e000084. doi:10.1136/esmoopen-2016-000084.

xxxi Lim SM, Park HS, Kim S, Kim S, Ali SM, Greenbowe JR, Yang IS, Kwon NJ, Lee JL, Ryu MH, Ahn JH, Lee J, Lee MG, Kim HS, Kim H, Kim HR, Moon YW, Chung HC, Kim JH, Kang YK, Cho BC. Next-generation sequencing reveals somatic mutations that confer exceptional response to everolimus. Oncotarget. 2016 Mar 1;7(9):10547-56. doi: 10.18632/oncotarget.7234.

xxxii Loaiza-Bonilla A, Jensen CE, Shroff S, Furth E, Bonilla-Reyes PA, Deik AF, Morrissette J. KDR Mutation as a Novel Predictive Biomarker of Exceptional Response to Regorafenib in Metastatic Colorectal Cancer. Cureus. 2016 Feb 3;8(2):e478. doi: 10.7759/cureus.478.

xxxiii Biswas R, Gao S, Cultraro CM, Maity TK, Venugopalan A, Abdullaev Z, Shaytan AK, Carter CA, Thomas A, Rajan A, Song Y, Pitts S, Chen K, Bass S, Boland J, Hanada KI, Chen J, Meltzer PS, Panchenko AR, Yang JC, Pack S, Giaccone G, Schrump DS, Khan J, Guha U. Genomic profiling of multiple sequentially acquired tumor metastatic sites from an "exceptional responder" lung adenocarcinoma patient reveals extensive genomic heterogeneity and novel somatic variants driving treatment response. Cold Spring Harb Mol Case Stud. 2016 Nov;2(6):a001263.

xxxiv Pietanza MC, Litvak AM, Varghese AM, Krug LM, Fleisher M, Teitcher JB, Holodny AI, Sima CS, Woo KM, Ng KK, Won HH, Berger MF, Kris MG, Rudin CM. A phase I trial of the Hedgehog inhibitor, sonidegib (LDE225), in combination with etoposide and cisplatin for the initial treatment of extensive stage small cell lung cancer. Lung Cancer. 2016 Sep;99:23-30. doi: 10.1016/j.lungcan.2016.04.014

xxxv Wen Y, Li H, Zeng Y, Wen W, Pendleton KP, Lui VW, Egloff AM, Grandis JR. MAPK1 E322K mutation increases head and neck squamous cell carcinoma sensitivity to erlotinib through enhanced secretion of amphiregulin. Oncotarget. 2016 Apr 26;7(17):23300-11. doi: 10.18632/oncotarget.8188.

xxxvi Lheureux S, Bruce JP, Burnier JV, Karakasis K, Shaw PA, Clarke BA, Yang SY, Quevedo R, Li T, Dowar M, Bowering V, Pugh TJ, Oza AM. Somatic BRCA1/2 Recovery as a Resistance Mechanism After Exceptional Response to Poly (ADP-ribose) Polymerase Inhibition. J Clin Oncol. 2017 Apr 10;35(11):1240-1249. doi: 10.1200/JCO.2016.71.3677.

xxxvii Gibson WJ, Ruan DT, Paulson VA, Barletta JA, Hanna GJ, Kraft S, Calles A, Nehs MA, Moore FD Jr, Taylor-Weiner A, Wala JA, Zack TI, Lee TC, Fennessy FM, Alexander EK, Thomas T, Janne PA, Garraway LA, Carter SL, Beroukhim R, Lorch JH, Van Allen EM. Genomic Heterogeneity and Exceptional Response to Dual Pathway Inhibition in Anaplastic Thyroid Cancer. Clin Cancer Res. 2017 May 1;23(9):2367-2373. doi: 10.1158/1078-0432.CCR-16-2154-T.

xxxviii Kollipara R, Schneider B, Radovich M, Babu S, Kiel PJ. Exceptional Response with Immunotherapy in a Patient with Anaplastic Thyroid Cancer. Oncologist. 2017 Oct;22(10):1149-1151. doi: 10.1634/theoncologist.2017-0096.

xxxix Knepper TC, Freeman ML, Gibney GT, McLeod HL, Russell JS. Clinical Response to Pazopanib in a Patient With KDR-Mutated Metastatic Basal Cell Carcinoma. JAMA Dermatol. 2017;153(6):607–609. doi:10.1001/jamadermatol.2017.0187

xl Dos Santos Fernandes G, da Motta Girardi D, Dib Batista Bugiato Faria L, Giacomini Bernardes JP, de Almeida Coudry R. Impressive response to immunotherapy in a metastatic gastric cancer patient: could somatic copy number alterations help patient selection? Journal for Immunotherapy of Cancer. 2017;5:84. doi:10.1186/s40425-017-0291-9.

xli Wilkes DC, Sailer V, Xue H, Cheng H, Collins CC, Gleave M, Wang Y, Demichelis F, Beltran H, Rubin MA,Rickman DS. A germline FANCA alteration that is associated with increased sensitivity to DNA damaging agents. Cold Spring Harb Mol Case Stud. 2017 Sep;3(5). pii: a001487. doi:10.1101/mcs.a001487.

xlii George S, Miao D, Demetri GD, Adeegbe D, Rodig SJ, Shukla S, Lipschitz M, Amin-Mansour A, Raut CP, Carter SL, Hammerman P, Freeman GJ, Wu CJ, Ott PA, Wong KK, Van Allen EM. Loss of PTEN Is Associated with Resistance to Anti-PD-1 Checkpoint Blockade Therapy in Metastatic Uterine Leiomyosarcoma. Immunity. 2017 Feb 21;46(2):197-204. doi: 10.1016/j.immuni.2017.02.001.

xliii Wander SA, Hasserjian RP, Oduro K, Glomski K, Nardi V, Cote GM, Graubert TA, Andrew M. Brunner AM, Chen YA, and Fathi AT. Combined Targeted Therapy for BRAF-Mutant, Treatment-Related Acute Myeloid Leukemia. JCO Precision Oncology 2017 :1, 1-7

xliv Patel M, Ikeda S, Pilat SR, Kurzrock R. JAK1 Genomic Alteration Associated With Exceptional Response to Siltuximab in Cutaneous Castleman Disease. JAMA Dermatol. 2017 May 1;153(5):449-452. doi: 10.1001/jamadermatol.2016.5554.

xlv Seim NB, Kang SY, Bhandari M, Jones RG, Teknos TN. Personalized Medicine Approach for an Exceptional Response to Multiple-recurrent and Metastatic HER2-positive Oropharyngeal Squamous Cell Carcinoma. Ann Otol Rhinol Laryngol. 2017 Apr;126(4):334-339. doi: 10.1177/0003489416687309.

xlvi Parachoniak CA, Rankin A, Gaffney B, Hartmaier R, Spritz D, Erlich RL, Miller VA, Morosini D, Stephens P, Ross JS, Keech J Jr, Chmielecki J. Exceptional Durable Response to Everolimus in a Patient with Biphenotypic Breast Cancer Harboring an STK11 Variant. Cold Spring Harb Mol Case Stud. 2017 May 26. pii: mcs.a000778. doi: 10.1101/mcs.a000778.

xlvii Rodríguez-Moreno JF, Apellaniz-Ruiz M, Roldan-Romero JM, Durán I, Beltrán L, Montero-Conde C, Cascón A, Robledo M, García-Donas J, Rodríguez-Antona C. Exceptional Response to Temsirolimus in a Metastatic Clear Cell Renal Cell Carcinoma With an Early Novel MTOR-Activating Mutation. J Natl Compr Canc Netw. 2017 Nov;15(11):1310-1315. doi: 10.6004/jnccn.2017.7018.

xlviii Sorscher S, Resnick J, Goodman M. First Case Report of a Dramatic Radiographic Response to a Checkpoint Inhibitor in a Patient With Proficient Mismatch Repair Gene Expressing Metastatic Colorectal Cancer. JCO Precision Oncology 2017 :1, 1-4 .

xlix Pishvaian MJ, Biankin AV, Bailey P, Chang DK, Laheru D, Wolfgang CL, Brody JR. BRCA2 secondary mutation-mediated resistance to platinum and PARP inhibitor-based therapy in pancreatic cancer. Br J Cancer. 2017 Apr 11;116(8):1021-1026. doi: 10.1038/bjc.2017.40.

l Korphaisarn K, Loree JM, Nguyen V, Coulson R, Holla V, Litzenburger BC, Chen K, Mills GB, Maru DM, Meric-Bernstan F, Mills Shaw KR, Kopetz S. Genomic analysis of exceptional responder to regorafenib in treatment-refractory metastatic rectal cancer: a case report and review of the literature. Oncotarget. 2017 Jun 3. doi: 10.18632/oncotarget.18357.
